# A Retrospective Study on the Efficacy, Safety, and Clinical and Radiological Outcomes of PEEK Anchors (CEPTRE® Knotted Suture Anchor and VIPLOK® Knotless Anchor) in the Treatment of Rotator Cuff Repairs

**DOI:** 10.7759/cureus.48632

**Published:** 2023-11-10

**Authors:** Vivek Pandey, Sandesh Madi

**Affiliations:** 1 Orthopaedics, Kasturba Medical College, Manipal, Manipal Academy of Higher Education, Udupi, IND

**Keywords:** anchor pullout, complications, peek anchor, suture anchor, rotator cuff repair

## Abstract

Background: Rotator cuff tears are a common cause of disability in the shoulder of the aging population. Rotator cuff repair is performed to address disability arising from rotator cuff tears, which fails to improve by conservative treatment. The present retrospective study was undertaken to explore the efficacy, safety, clinical, and radiological outcomes of PEEK suture anchors (Ceptre® suture anchor and Viplok® knotless anchor; Sironix, Healthium MedTech, India) in the treatment of arthroscopic rotator cuff repairs.

Method: This is a retrospective cohort study of arthroscopic rotator cuff repair of posterosuperior tears, which was performed between January 2019 and December 2020 with a minimum follow-up of one year. The demographic data, history, preoperative clinical, magnetic resonance imaging report, and intraoperative details of each patient operated on were obtained from medical records. Post-operative clinical assessments of patients were done based on American Shoulder and Elbow Score (ASES) and Single Sssessment Numeric Evaluation (SANE) scores. Ultrasonography (USG) was done to assess any anchor pull-out and structural healing of the cuff. X-rays were performed to look for the anchor’s metallic tip migration.

Results: A total of 65 patients were included in the study. There were 43 males (66.2%) and 22 females (33.8%). The mean age was 55.98 (±7.9) years (range: 40-69 years). The mean follow-up was 21.9 months (range: 12-46). Mean (±SD) postoperative ASES and SANE scores at the end of one year were 81.5 (±14.27) and 83.55 (±13.83), respectively. There was significant improvement with respect to the preoperative scores (p<0.0001). USG revealed complete healing in 80% of cases, partial tears in 10.8 %, and full-thickness tears in 9.2% of cases. No anchor pull-outs were noted on USG. X-rays did not reveal unusually large cystic areas in the humeral head or metallic tip migration from the humeral head at their last follow-up.

Conclusions: The results of this study suggest that PEEK suture anchors (Ceptre® knotted suture anchor and Viplok® knotless anchor; Sironix, Healthium MedTech, India) are a viable option for successful surgery in the treatment of arthroscopic rotator cuff repairs without any major anchor related complications.

## Introduction

Rotator cuff tears are increasingly identified as a source of shoulder pain and disability, especially in an aging population [1]. The treatment includes conservative and operative options. The clinical decision-making regarding conservative vs. operative could be quite complex as robust and high-quality evidence lacks definitive indications for surgical intervention [2,3]. In case of an operative decision, repairing the torn cuff back to its native footprint appears to be the current standard of care for a full-thickness cuff tear. Appropriate knowledge of pathology and healing patterns of the cuff, strong and biological repair techniques, better suture anchors, and gradual post-cuff repair rehabilitation have led to good to excellent outcomes after repair [4]. Repairing the cuff onto the footprint is performed by using suture anchors or a transosseous technique [5]. Although both techniques result in similar clinical and radiological outcomes [6], techniques deploying suture anchors are far more popular than transosseous ones.

Suture anchors are made of different materials, and surgeons must understand their indications and limitations. The characteristics of an ideal suture anchor would be easy to handle, strong pullout strength, minimal soft tissue and bone reaction, able to perform its designed function as long as needed, and dissolve, leaving no trace [7]. In addition to these characteristics, it is desirable that the anchors used to repair soft tissues do not interfere with post-operative/follow-up imaging with ultrasonography (USG) or magnetic resonance imaging (MRI). Based on the biomaterial characteristics, Cho et al. classified suture anchors as metallic, biodegradable, biostable (polyetheretherketone, PEEK), biocomposite (β-TCP), and all-suture (soft) anchor types [8]. A PEEK anchor is made of a polymer obtained by dialkylation of bisphenol. PEEK anchors are classified under the biostable class due to their ability to resist in vivo degradation and biomechanical properties (in contrast to biodegradable or biocomposite materials) [8]. Several studies in the past have tested the cytotoxicity, immunogenic, and mutagenic response using PEEK implants and concluded that PEEK biomaterials are effectively inert [9]. Further, PEEK polymers exhibit elastic modulus similar to cortical bone [10]. Equally good results in terms of pullout strength, initial load to failure, and cyclic loading have been shown in several biomechanical studies comparing bio-resorbable poly-L-lactic acid (PLLA) anchors and non-resorbable PEEK anchors [11]. Based on these properties, PEEK materials offer superior fixation properties without an adverse local reaction from the degradation of polymers. Since the 1990s, PEEK-based orthopedic implants have been widely used in various spine and trauma procedures. However, there is a paucity of literature on the clinical and radiological implications of using PEEK suture anchors in rotator cuff repairs [12-14].

The present retrospective study was undertaken to explore the efficacy, safety, and clinical and radiological outcomes of PEEK suture anchors (Ceptre® suture anchor and Viplok® knotless anchor; Sironix, Healthium MedTech, India) in the treatment of all-arthroscopic rotator cuff repairs.

## Materials and methods

Study design and patient selection: This is a retrospective cohort study of arthroscopic rotator cuff repair of posterosuperior tears, which was performed between January 2019 and December 2020 with a minimum follow-up of one year. The study was initiated after the approval of the Institutional Ethical Committee, Kasturba Medical College, Manipal (IEC/194/2022).

The demographic data, history, preoperative clinical, preoperative magnetic resonance imaging (MRI) report, and intraoperative details of each patient operated on were obtained from offline and online medical records. Seventy-seven patients who matched our criteria were selected. A total of 11 patients were lost to follow-up, leaving 65 patients for the final assessment.

Inclusion and exclusion criteria: The inclusion criteria were (1) patients between 40 years and 70 years, (2) the presence of full-thickness posterosuperior cuff tear confirmed during arthroscopy, (3) repair technique either by single row (SR) or double row suture bridge (DRSB), (4) post-operative x-ray and ultrasonographic imaging of repaired cuff at 12 months, and (5) final clinical follow-up at a minimum of 12 months. The exclusion criteria were (1) Grade 3 supraspinatus muscle atrophy of occupation ratio of less than 0.4 (Thomazeau classification) [15], (2) Grade 4 fatty infiltration of supraspinatus and infraspinatus (Goutallier classification) [16], (3) Osteoporotic humeral head, (4) preoperative cyst in the humeral head observed on MRI, (4) mini-open repair of the cuff, (5) irreparable rotator cuff, (6) partial repair of the cuff, and (7) repair using anchors made of material other than PEEK.

Operative technique: All cases were operated by a single senior surgeon with experience of more than 10 years in performing rotator cuff repair. All patients were operated on under general anesthesia and interscalene block in sloppy lateral decubitus position with the affected upper limb attached to the limb positioner (Spider 2 limb positioner, Smith & Nephew, Andover, MA). After standard skin preparation and draping, diagnostic arthroscopy of the affected shoulder joint was performed from the standard posterior portal. The anterior portal was made just above the subscapularis tendon in the rotator interval. The biceps tendon was tenotomized if it was significantly frayed, flat, split, or unstable due to pulley damage. Tenodesis was performed if the patient had demanded it preoperatively, in a manual laborer, or when the patient was less than 50 years old. Lafosse Type 1 subscapularis tendon was debrided, whereas all other types were repaired with one or two anchors.

After evaluating the glenohumeral joint, the scope was shifted to the subacromial space. Standard subacromial bursa excision was done using a power shaver and radiofrequency device. Following the bursa excision, supra- and infraspinatus tendons were assessed for the following characteristics (shape, size, retraction, and reparability onto the footprint) before the repair. Tear size in the anteroposterior direction was measured intraoperatively using a graduated probe and categorized using the DeOrio and Cofield classification [17]. If the cuff was found to be retracted, the standard releases were performed until optimum footprint coverage (>80%) was obtained. Apical traction suture was applied in the case of an ‘L or reverse L’ shape cuff tear for traction while releasing the tendon from adhesions or para labral capsule. The sclerosed bone over the greater tuberosity was gently dusted till minimal bleeding ensued.

Repair technique: The cuff was repaired either with SR or DRSB technique, which was dependent on the size and repairability of the tear. Small size tears were repaired with the SR technique, whereas medium size and above were repaired with the DRSB technique. In the SR technique, one or two double-loaded suture anchors (4.8 mm Ceptre PEEK anchor; Sironix, Healthium MedTech, India) were placed in the middle of the greater tuberosity. The cuff was repaired using the modified Mason-Allen technique [18]. In the DRSB technique, two to three were used for the medial row and were inserted just lateral to the cartilage margin. Mattress bite was taken in the cuff just lateral to the musculotendinous junction. In case of an L- or reverse-L-shape tear, one or two side-to-side intratendinous sutures were placed and tied. Once sutures were passed, they were tied in a mattress fashion. Three to four single suture limbs were pulled out of the cannula and passed via the eyelet of the lateral row knotless anchors (4.75 mm Viplok® knotless PEEK anchor; Sironix, Healthium MedTech, India), followed by anchor insertion onto the lateral side of the humerus, 1 cm below the greater tuberosity, to create a suture bridge construct (transosseous equivalent). Bony acromioplasty was performed using a motorized burr if there was an acromial spur or Bigliani type III acromion. All the intraoperative findings were recorded in a standardized form.

Intraoperative complications pertaining to suture anchor: Complications such as anchor breakage, pullout, and suture strand breakage were noted in the operative notes.

Post-operative rehabilitation: All the patients were started on a structured rehabilitation protocol. Post-operatively, the shoulder was immobilized in an arm sling for four weeks, and only elbow and finger movements were encouraged, along with scapular isometrics. After four weeks, passive mobilization of the shoulder was started. At the end of eight weeks, active assisted movements were initiated, followed by active movements. At the end of three months, all patients underwent USG of the shoulder to determine the healing status of the rotator cuff over the footprint. Further, cuff strengthening exercises were initiated with TheraBand. Return to full activity and sports activity was reserved at the end of 8-12 months.

**Figure 1:**
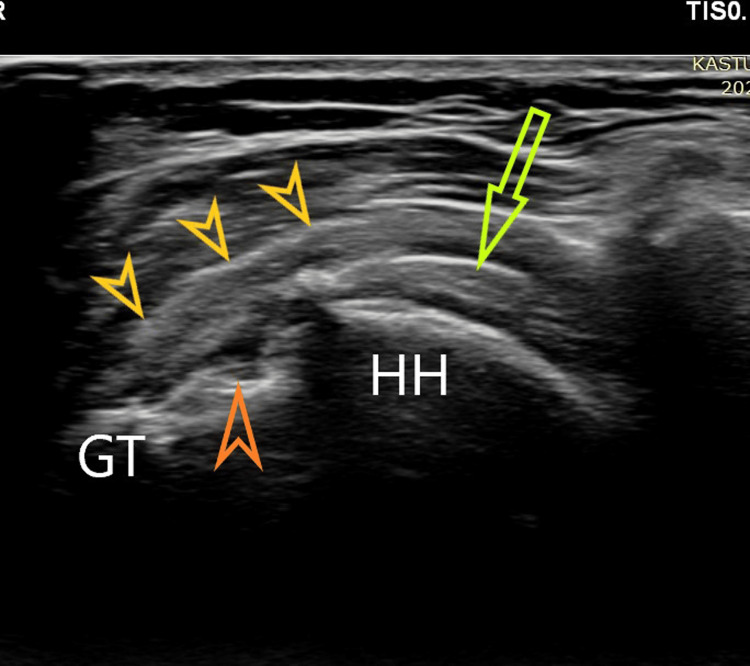
Postoperative ultrasonographic image of a repaired rotator cuff. The yellow arrow indicates the repaired cuff on greater tuberosity, the orange arrow indicates the tip of the anchor inserted on the tuberosity, and the green arrow indicates sutures inside the rotator cuff substance. GT, greater tuberosity; HH, Humeral head

Post-operative rotator cuff integrity and anchor pullouts: The assessment of the healing status of the repaired tendon was performed using an ultrasound examination at the end of three months and then yearly. USG was performed on Philips epic 5G (Philips Medical Systems International B.V., Best, Netherlands) with a linear probe (12-5 MHz) by a single qualified senior musculoskeletal radiologist (Figure 1). Based on USG, the integrity of the repaired cuff was classified broadly into three categories: Type I, normal thickness with homogeneously hyperechoic tendon or partial hypoechogenicity or heterogenicity or insufficient thickness without discontinuity, indicating ‘complete healing’; Type II, the presence of a minor discontinuity or a focal partial defect, indicating ‘partial tear’; and Type III, the presence of a major discontinuity or a ‘full-thickness tear’. Recently, Gartsman et al. and Gwark et al. also deployed similar criteria for ultrasound assessment of the post-operative healing status of the cuff [19,20]. Gilat et al. proved that the US showed a sensitivity of 80.8% and specificity of 100% in diagnosing rotator cuff retear [21]. The anchor pullouts in the subacromial space were detected with the same. Martinel et al. have used the same technique to assess anchor pullout in a series of 102 patients [22].

**Figure 2:**
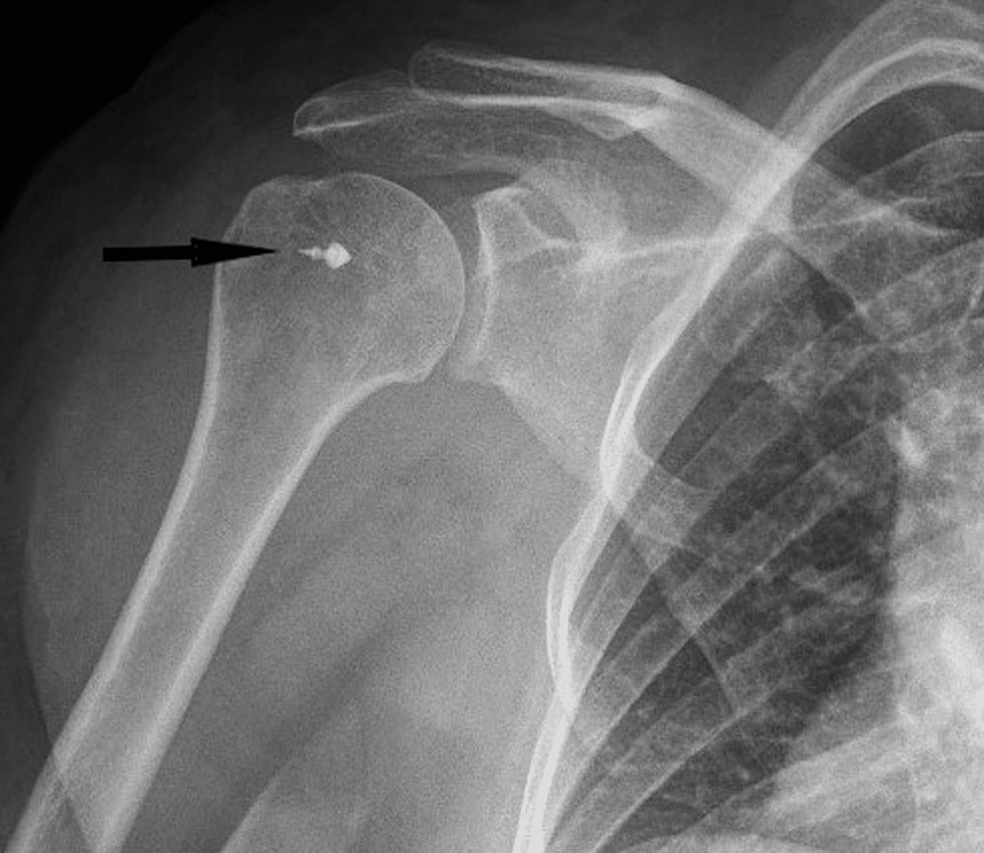
Postoperative plain radiograph of the shoulder showing a bullet tip (of lateral row anchor) in situ (black arrow).

Post-operative plain radiograph of the shoulder: A plain x-ray was performed at the one-year and/or last follow-up to note any Viplok bullet (metallic tip) pullout (Figure 2).

Functional outcome analysis: At a minimum follow-up of one year, an independent assessor assessed the functional outcome with an American Shoulder and Elbow Score (ASES) and Single Assessment Numeric Evaluation (SANE) [23,24].

Statistical analysis: Statistical analysis was performed using Statistical Product and Service Solutions (SPSS, version 16.0) (IBM SPSS Statistics for Windows, Armonk, NY). Descriptive analysis was performed to assess the various demographic factors. The data were presented as the mean and standard deviation for continuous variables and percentages for the categorical variables. Paired t-test was used to compare the paired means. The p-value of <0.05 was considered significant.

## Results

A total of 65 patients were included in the study. There were 43 males (66.2%) and 22 females (33.8%). The mean age (±SD) was 55.98 (±7.9) years (range: 40-69 years). The right side was involved more (64.6%, n=42) than the left (34.4%, n=23). The mean follow-up was 21.9 months (range: 12-46). Mean (±SD) preoperative ASES and SANE scores were 41.32 (±5.1) and 38. 96 (±4.7), respectively. Other baseline data regarding tear characteristics and operative procedures are mentioned in Table 1.

**Table 1:** Intraoperative characteristics of the rotator cuff, procedure performed, and rotator cuff healing on USG. Supraspinatus muscle atrophy is graded as per the Thomazeau classification. Fatty infiltration of muscles is as per the Goutallier classification.

Characteristics		Number (percentage)	P-value
Posterosuperior tear size	Small	5 (7.7%)	0.002
	Medium	19 (29.2%)	
	Large	15 (23.07%)	
	Massive	26 (40%)	
Shape	Crescent	24 (36.9%)	0.0003
	L/reverse L	25 (38.4%)	
	U shape	12 (18.46%)	
	Unclassified	4 (6.15%)	
Subscapularis tear	None	22 (33.84%)	0.0012
	Type 1	14 (21.53%)	
	Type 2	4 (6.15%)	
	Type 3	17 (26.15%)	
	Type 4	6 (9.23%)	
	Type 5	2 (3.07%)	
Muscle atrophy supraspinatus	Grade 0	48 (73.8%)	0.0001
	Grade 1	17 (26.2%)	
Fatty infiltration supraspinatus	Stage 0	26 (40%)	0.167
	Stage 1	21 (32.3%)	
	Stage 2	14 (21.53%)	
	Stage 3	4 (6.15%)	
Fatty infiltration infraspinatus	Stage 0	24 (36.9%)	0.006
	Stage 1	23 (35.38%)	
	Stage 2	15 (23.07%)	
	Stage 3	3 (4.61%)	
Biceps procedure	None	30 (46.15%)	0.012
	Tenotomy	24 (36.92%)	
	Tenodesis	11 (16.9%)	
Acromioplasty	None	61 (93.84%)	<0.0001
	Yes	4 (6.15%)	
Type of repair	SR	16 (24.61%)	<0.0001
	DRSB	49 (75.38%)	
Rotator cuff healing on USG	Type 1	52 (80%)	<0.0001
	Type 2	7 (10.8%)	
	Type 3	6 (9.2%)	

Regarding anchor-related complications, there were no incidences of intraoperative anchor breakages during insertion. In two cases, the medial row, 4.8 mm, PEEK Ceptre® suture anchor got pulled out due to underlying osteoporosis. The pulled-out anchor was replaced with 6.5 mm titanium anchors, and these cases were excluded from the study. In two cases, the lateral row Viplok® knotless anchor also got pulled out and replaced with another company anchor. There were no incidences of lateral row anchor pullout or breakages. No instances of suture breakdown were detected while tying the knots. Both the anchors (medial row PEEK and lateral row knotless) were found to be easy to deploy.

Mean (±SD) postoperative ASES and SANE scores at the end of one year were 81.5 (±14.27) and 83.55 (±13.83), respectively. There was significant improvement with respect to the preoperative scores (p<0.0001) (Table 2).

**Table 2:** Pre- and post-operative ASES and SANE scores of the patients. ASES, American Shoulder and Elbow Score; SANE, Single Assessment Numeric Evaluation

	Mean (± SD) preoperative score	Mean (± SD) postoperative score	P-value
ASES score	41.32 (± 5.1)	81.5 (±14.27)	< 0.0001
SANE score	38. 96 (± 4.7)	83.55 (±13.83)	< 0.0001

The sonographic evaluation revealed complete Type 1 healing in 80% (n=52), Type II in 10.8 % (n=7), and Type III in 9.2% (n=6) of cases. All Type 2 and Type 3 tears occurred in large (Type 2 - 2; Type 3 - 2) and massive size (Type 2 - 5; Type 3 - 4) tear repair. Furthermore, USG did not detect any anchor pullout in the subacromial space. No bullet tip migration (out of humerus) was noted in the case of lateral row anchor. No local skin complications, such as allergic reactions, were noted. There were no superficial or deep infections in any of the patients.

## Discussion

The current study aimed to investigate the efficacy, safety, and clinical and radiological outcomes of PEEK suture anchors (medial knotted and knotless lateral row anchors - Ceptre® knotted suture anchor and Viplok® knotless anchor; Sironix, Healthium Medtech, India) in the treatment of arthroscopic rotator cuff repair. The findings of this study demonstrated a high success rate of the rotator cuff repair procedure, with significant improvements in functional outcomes without any anchor-related complications. These results are consistent with previous research in the field, supporting the effectiveness of PEEK anchors in rotator cuff repair to restore shoulder function.

The success of rotator cuff repair depends on numerous patient and surgical factors. Some of the technique-related factors include adequate mobilization of the retracted tendons, preparation of the footprint, proper placement of the suitable suture anchors, suture bites placed just lateral to the musculotendinous junction, and adequate compression of the tendon over footprint by suture fixation [4]. Single-row and double-row suture bridges are the two most commonly employed fixation techniques, and the decision is primarily based on the cuff tear characteristics and the surgeon's experience. A double-row technique has improved footprint coverage and initial fixation strength compared to a single row [25]. Both these fixation techniques were accomplished using PEEK suture anchors exclusively in our study without any adverse intra-operative issues, such as an anchor or suture breakage or postoperative anchor pull-out. Two medial and lateral row anchors got pulled out after their insertion, probably due to underlying osteoporosis.

In our study using PEEK anchors, the mean (+SD) preoperative and postoperative ASES scores were 41.32 (±5.1) and 81.5 (±14.27), respectively, at a minimum one-year follow-up. In a prospective study by Lapner et al., the preoperative and postoperative ASES scores (+SD) were 50.8 (±18.4) and 85.7 (±17.8), respectively, at one-year follow-up [26]. In a retrospective study, Assunção et al. recruited 143 patients, and the authors reported a postoperative ASES score of 81.2 (±20.8) at two years of follow-up [27]. In another retrospective study by Gao et al., the post-operative ASES score was (82.5±11.5) at a one-year follow-up [28]. In a study by Pandey et al., the mean ASES score at a five-year follow-up was approximately 87 (for SR and DRSB technique) [29]. Therefore, the functional outcome data from various clinical studies are in concordance with our findings.

Further in our study, the mean (+SD) preoperative and postoperative SANE scores were 38.96 (±4.7) and 83.55 (±13.83), respectively, at a minimum one-year follow-up after surgery. In a prospective randomized study by Burks et al., the preoperative and postoperative SANE scores were 40.8 (±22.2) and 90.4 (±15.9), respectively, at one-year follow-up [30]. A similar improvement in the postoperative SANE score of 94 (±10.26) was reported in a study involving elderly patients with arthroscopic repair of traumatic rotator cuff tears [31]. In a retrospective review, Mijic et al. recruited 535 patients, and the authors reported mean preoperative and postoperative SANE scores of 45.7 and 83.2, respectively [32]. The SANE scores of various studies are similar to our data.

In our study, the postoperative anchor pull-out was assessed by USG. USG was performed at three and 12 months, which did not reveal pull-outs of any of the medial or lateral row anchor anchors at either of the timelines. A similar technique has been used by Martinel et al. [22]. USG has been advocated as a helpful tool for assessing anchor placement [33]. Plain radiographs can be utilized to assess anchor migration in the case of metal anchors. However, they are of no use in assessing PEEK anchor migration. In our study, we assessed the bullet tip migration (of knotless lateral row anchor) and did not notice any tip migration out of the bone at one year or the last follow-up.

Both MRI and USG have been used for the postoperative integrity of the repaired rotator cuff tendon. Lee et al. reported MRI and US to be comparable in diagnosing the postoperative full-thickness retear; both carry lower sensitivity for partial tears [34]. In a recent systematic review, Gyftopoulos et al. reported similar mean sensitivity-specificity for MRI and USG at 81.4%-82.6% and 83.7%-90.7%, respectively [35]. In our study, the sonographic evaluation revealed complete Type 1 healing in 80% (n=52), Type II in 10.8% (n=7), and Type III in 9.2% (n=6) of cases. All Type 2 and Type 3 tears occurred in large and massive tear repair. In a retrospective study using HEALIX anchors (DePuy Synthes, Raynham, MA) conducted by Aguado et al. in 90 patients, the sonographic evaluation revealed Type I complete healing in 74.5% (n=67), Type II partial healing in 11.1% (n=10), and Type III healing (complete full thickness tear) in 14.4% (n=13) patients [36]. Using Arthrex PEEK anchors (Arthrex Manufacturing, Inc., Naples, FL), at a five-year follow-up, Pandey et al. reported overall Type 2 and 3 retear rates of 17.8% and 4.8%, respectively [29]. These studies report similar retear rates of rotator cuff with standard techniques and anchors.

PEEK anchors were developed over biodegradable anchors, given complications such as anchor breakage during insertion and potential inflammatory response resulting in the formation of perianchor cysts and associated intraarticular granuloma. Being biologically inert with robust mechanical properties and easy to mold, the prospect of PEEK materials as suture anchors appeared promising. However, even with PEEK anchors, the formation of perianchor cysts has been noted. Ro et al. evaluated the differences in the perianchor bone reaction in patients who underwent rotator cuff tear repairs with all-suture, bioabsorbable screw, and PEEK-type suture anchors [37]. They found that the proportions of perianchor bone reaction for grade 2 (Kim et al.'s classification [38]) and above (indicating perianchor bone cysts) were 8.8%, 12.5%, and 16.7% in the all-suture, PEEK, and bioabsorbable-type anchor groups, respectively (p=0.485). Nevertheless, the retear rate was associated with a greater perianchor cystic reaction (p=0.001). In contrast to this observation, Kim et al. noted an unexpectedly high rate of fluid formation (41.7%) with PEEK anchors compared with the all-suture anchors at one year of cuff surgery [39]. However, the authors have not stated any reason for this unusual observation. Interestingly, no relationship was observed between the presence of perianchor fluid and the integrity of the rotator cuff repair. We could not assess perianchor lysis or cyst formation as the postoperative rotator cuff assessment was performed with USG.

Furthermore, it has been noted that biochemical (anchor material) factors and biomechanical factors, such as tear size, degree of retraction, number of anchors, and varying bone density within the humeral head, are responsible for developing these perianchor cysts [40]. After double-row rotator cuff repair, Haneveld et al. compared the osseous reaction between bioabsorbable poly-L-lactic acid (PLLA) and PEEK anchors [11]. They found that, regardless of the anchor material, enhanced anchor tunnel widening was noted in the lateral anchor row compared to the medial row, highlighting mechanical stress as a factor for developing perianchor fluid and tunnel widening. However, our study could not ascertain such observations due to a lack of postoperative MRI. Although the postoperative radiographs at a minimum of one year or the last follow-up did not show any significant lucency in the bone, it is difficult to quantify it.

Even though the perianchor cysts are noted in PEEK anchor use, the biological effect on cuff healing and subsequent clinical outcomes appears to be largely unaffected. In a study by Lee et al., the functional and radiologic outcomes of PEEK and biocomposite (PLLA/poly(lactic-co-glycolic acid) 70% + β-tricalcium phosphate) anchors were evaluated, especially in terms of perianchor cyst formation during the first three and six months postoperatively [14]. Although the PEEK anchor had a significantly lower grade of perianchor cyst formation during the third postoperative month of follow-up, the degree of cyst formation was similar in both anchors at six months. Despite the initial increase in fluid collection during the early stages of rotator cuff healing, caused by the formation of perianchor cysts around the biocomposite anchor, these cysts gradually diminished over time. Importantly, this reduction in perianchor cysts did not have any detrimental effects on the integrity of the repaired rotator cuff or the functional outcomes of patients within the first six months [14]. The authors postulate that the high prevalence of perianchor fluid collection in the early phase of repair is probably due to anchor micromotion from the tensile load of the repaired rotator cuff [14]. Further, this reaction decreases with time depending on bony ingrowth, repair site stability, and fluid resorption. Similar observations were made by Micic et al., who also concluded that non-absorbable PEEK anchors had lower osteolysis rates than biodegradable anchors, and the retear rates were not significantly affected by the presence of osteolysis [41]. In the index study, the osteolysis around the PEEK anchors was looked up using plain radiographs at one-year follow-up, and no case of unusual osteolysis of the humeral head was noted.

A criticism of using PEEK materials is its poor bone integration attributed to its hydrophobic and unabsorbable properties. Hence, revision surgery with PEEK anchors in situ can be complicated in cases of large tears that are likely to retear. Recently, there have been specific design changes in the PEEK anchors to overcome this concern, such as vented open-construct PEEK anchors to encourage bone integration through these vents. Kim et al. performed a prospective randomized control study to compare the clinical and radiological outcomes between open-construct PEEK anchors versus non-vented biocomposite anchors [42]. At six months postoperatively, the status of bone ingrowth into the anchor and the presence of cyst formation were evaluated by computed tomography scan using the modified Barber’s ossification scale [43]. Better bone in-growth was noted with open construct PEEK anchors, but similar improvements were observed in functional outcomes, perianchor cyst formation, and retear rates in both groups. Chahla et al. conducted a randomized controlled study comparing the bony ingrowth of a coil-type open architecture anchor to that of a solid screw-type PEEK anchor for medial row fixation. The study found that both anchors exhibited similar levels of superior bone ingrowth [12]. Although this observation seems to be a significant improvement in the design of PEEK anchors, whether this osseointegration translates to improved biomechanical fixation strength and reduced retear rates needs further evaluation. Nevertheless, it is essential to highlight that, despite having similar clinical outcomes and complication rates in open-vented design compared to conventional solid designs, open-vented PEEK designs are more expensive than solid ones. In the index study, the PEEK anchors used for the medial and lateral rows were non-vented solid anchors.

Recently, Gao et al. conducted a multicentric, prospective, single-blind, randomized controlled trial to evaluate PEEK anchors manufactured by two companies to compare functional outcomes and complications after rotator cuff repair [28]. The authors observed that PEEK anchors from both companies performed equally well without any significant complications at the six-month follow-up. Further, the authors noted that a cheaper PEEK anchor would be more suitable in developing countries, especially where many of the population do not have insurance coverage [28].

Although in this study, no direct comparisons were made between PEEK anchors produced by different companies, the performance of indigenously manufactured PEEK anchors (CEPTRE® knotted suture anchor and VIPLOK® knotless anchor) in treating posterosuperior rotator cuff tears was found to be comparable to other anchors. Furthermore, these anchors demonstrated satisfactory outcomes without anchor-related complications, such as intraoperative anchor breakages, suture thread breakage, or postoperative anchor pull-out at the minimum one-year follow-up period. Furthermore, no adverse events or allergic reactions were reported by any patients during the follow-up.

Limitations of the study: Despite the positive outcomes, it is vital to acknowledge the limitations of this study. The major limitation is the lack of postoperative MRI assessment, which led to a lack of conclusion regarding cystic or perianchor lytic changes in the humeral head. Secondly, though USG did not detect any subacromial pull-out of the anchors, MRI could have been a better investigation to detect the same. Lastly, biases in retrospective studies, such as information, selection, or recall bias, may affect the outcome.

## Conclusions

In conclusion, this study contributes to the growing body of evidence supporting the effectiveness of rotator cuff repair in improving shoulder function using PEEK suture anchors (Ceptre® knotted suture anchor and Viplok® knotless anchor; Sironix, Healthium MedTech, India) without any significant complications. The results of rotator cuff repair using these PEEK anchors are similar to other anchors and do not display any untoward complications.
